# Development and Validation of an Evaluation Tool to Measure the Effectiveness of a Smoking Cessation Training among Healthcare Providers in Malaysia: The Providers’ Smoking Cessation Training Evaluation (ProSCiTE)

**DOI:** 10.3390/ijerph16214297

**Published:** 2019-11-05

**Authors:** Siti Idayu Hasan, Farizah Mohd Hairi, Amer Siddiq Amer Nordin, Mahmoud Danaee

**Affiliations:** 1 Department of Social & Preventive Medicine, Faculty of Medicine, University of Malaya, Lembah Pantai, 50603 Kuala Lumpur, Malaysia; ayu_umcas@um.edu.my (S.I.H.); mdanaee@um.edu.my (M.D.); 2 Nicotine Addiction Research Group UMCAS, Wisma R & D University of Malaya, Jalan Pantai Baharu, 59200 Kuala Lumpur, Malaysia; amersiddiq@um.edu.my; 3 Department of Psychological Medicine, Faculty of Medicine, University of Malaya, Lembah Pantai, 50603 Kuala Lumpur, Malaysia

**Keywords:** content validity, construct validity, exploratory factor analysis, healthcare providers, program evaluation, smoking cessation, training, 5 As brief intervention

## Abstract

**Background**: In line with Article 14 of the Framework Convention for Tobacco Control, we have witnessed vast developments in smoking cessation training for healthcare providers, offering help for smokers. However, there is no specific evaluation tool to monitor and evaluate the effectiveness of these programs for future enhancement and sustainability. **Objective**: To develop and validate a new tool for evaluating smoking cessation training programs for healthcare providers called the Providers’ Smoking Cessation Training Evaluation (ProSCiTE). **Methods**: The 74-item ProSCiTE tool was developed based on a review of the literature and an expert panel review. The tool was validated in a sample of 403 healthcare providers using a cross-sectional study design from July to December 2016. Content validity was assessed by the Scale-Content Validity Index (S-CVI). The construct validity of the ProSCiTE was analyzed using exploratory factor analysis (EFA) to confirm psychometric properties. Internal consistency reliability was determined using Cronbach’s alpha. **Results**: The content validity showed that the S-CVI ranged from 0.82 to 1.00 for consistency, representativeness, relevancy, and the clarity of each construct, resulting in 67 items for the questionnaire. The construct validity of the ProSCiTE (based on eigenvalues and factor loadings to confirm the four-factor structure (attitude, self-efficacy, behavior, and barriers) with 54.74% total variance) was acceptable (Kaiser-Mayer-Olkin = 0.923; Bartlett’s test of sphericity was significant, *p* < 0.001). The internal consistency reliability of the four-factor structure was very good, with Cronbach’s alpha values at 0.89, 0.94, 0.95, and 0.90, respectively. **Conclusions**: This study showed that 67 items of the ProSCiTE demonstrated good content and construct validity, as well as a high internal consistency reliability for the measurement of knowledge, attitudes, self-efficacy, behavior, and barriers to smoking cessation interventions among healthcare providers. Therefore, the ProSCiTE is a valid and reliable research tool with which to evaluate the effectiveness of smoking cessation training programs.

## 1. Introduction

A valid and reliable tool is a very important key indicator to evaluate the effectiveness of a program. In order to evaluate the impact of a program, particularly to assess the effects of smoking cessation training on a 5 As (ask, advice, assess, assist and arrange) brief intervention, a good and valid tool is essential. Currently, to the best of our knowledge, there is no valid and reliable tool to evaluate healthcare providers’ smoking cessation training through examining their psychometric properties. Only a few questionnaires were found from previous studies worldwide that measured the effectiveness of smoking cessation training. Some studies showed promising results, but most were not specific or not standardized, and they did not measure any content validity or psychometric properties [1,2,3,4,5,6,7,8]. 

Therefore, we developed and tested an evaluation tool called “Providers’ Smoking Cessation Training Evaluation”, or in short, ProSCiTE, within our population. This quantitative study provides an empirical dataset based on healthcare providers in Malaysia. The tool is based on the 5 As brief smoking cessation intervention model, which is recommended by the Malaysian Clinical Practice Guidelines on the Treatment of Tobacco Use Disorder [9] and the U.S. Public Health Service Clinical Practice Guidelines [10,11]. This model is based on five strategies (asking about tobacco use, advice to quit, assessing willingness to make a quitting attempt, assisting in quitting attempt, and arranging for follow-up) and has been effective in both research and clinical practices. The 5 As approach has been shown to have an impact on treating smokers, particularly in the adult population [11,12,13]. A review by Cochrane showed that increasing the amount of behavioral support by healthcare providers likely increases the chances of success by about 10%–25% [12]. Both these guidelines recommend that all healthcare providers deliver a brief evidence-based intervention such as the 5 As to nicotine-dependent patients on each visit: 5 As provides a concise guideline for clinicians’ decision-making at points of care in a smoking cessation intervention. 

Despite clear recommendations and evidence, healthcare providers currently only provide a limited amount of support to their patients on smoking cessation due to several factors that differ across groups of healthcare providers [6,14,15,16,17,18,19,20,21,22,23]. These factors include uncertainty on how to implement brief interventions or a lack of familiarity with nicotine dependence assessments and effective treatments [24]. Previous studies have identified a number of barriers for the delivery of tobacco use interventions, including lack of training [25]; competing priorities and a mythological belief that counseling is not an appropriate service [26]; lack of time, manpower, and finance; lack of skills; concern for the clinician–patient relationship; the perception of motivation insufficiency in patients; and low intervention rates [27,28,29]. Healthcare providers have also claimed that they lack knowledge in smoking cessation counseling techniques and lack confidence in the smoking cessation program [30]. According to previous studies, the most significant barrier in providing smoking cessation intervention is the limited training of healthcare providers [31,32,33]. 

Healthcare providers, as front-liners in facilitating smoking cessation, are in an ideal position because they have frequent contact with patients. They are also the most trustworthy people to rely on for health purposes and are considered role models by patients [34,35]. Despite this significant evidence about the role of healthcare providers, in one study, only 9.7% of patients visited healthcare providers out of the 50% of patients that wanted to quit [36]. The U.S. Centers for Disease Control and Prevention reported that almost three-quarters of patients would like to stop smoking [37]. However, less than 5% of patients who quit smoking on their own remain abstinent for one year [38]. An analysis of 17 trials investigating physicians’ advice as an intervention discovered that even brief advice was effective in increasing the odds ratio for quitting smoking (1.74) [12]. This evidence shows that patients require assistance from healthcare providers to quit smoking successfully. Thus, in order to treat smokers, healthcare providers need to be trained and prepared to be more competent with both pharmacological and behavioral therapy skills. 

Training plays a crucial part in healthcare services’ development and sustainability. It fortifies healthcare providers to acquire new skills, strengthen their existing skills, increase productivity, as well as advance all healthcare providers to a higher level with similar skills and knowledge. Training programs around the world have been shown to have a significant impact on nicotine dependence treatment initiatives delivered by healthcare providers [2,7,8,32,39,40,41,42,43,44,45,46,47,48,49,50,51,52,53]. Therefore, it is paramount to examine and evaluate the effectiveness of these programs. This is due to the fact that these training programs may influence policymakers’ decision-making on the sustainability and improvement of the overall smoking cessation program. 

Several tools have been examined to measure the effectiveness of training [1,2,3,4,5,6,7,8,54,55,56]. Yet, researchers examining smoking cessation training programs cannot assume that these tools measure each program equally well. Even though some studies have explored usable constructs for evaluating smoking cessation training programs, continuous research is needed among healthcare providers at various stages of the 5 As intervention. The extent to which the implementation of this guideline should be reviewed and scrutinized is to ensure that the potential maximum impact is achieved. Whether knowledge and skills from the training have been applied in real practice is still doubtful.

The present study aimed to develop and validate the ProSCiTE tool for smoking cessation training. The measures hypothesized to be associated with healthcare providers’ practices in terms of smoking cessation are discussed in this article. This study investigates the content validity, construct validity, and reliability of the ProSCiTE. We also identify how these factors interact and potentially predict the implementation of each component of the 5 As smoking cessation model.

## 2. Materials and Methods 

**Figure 1:**
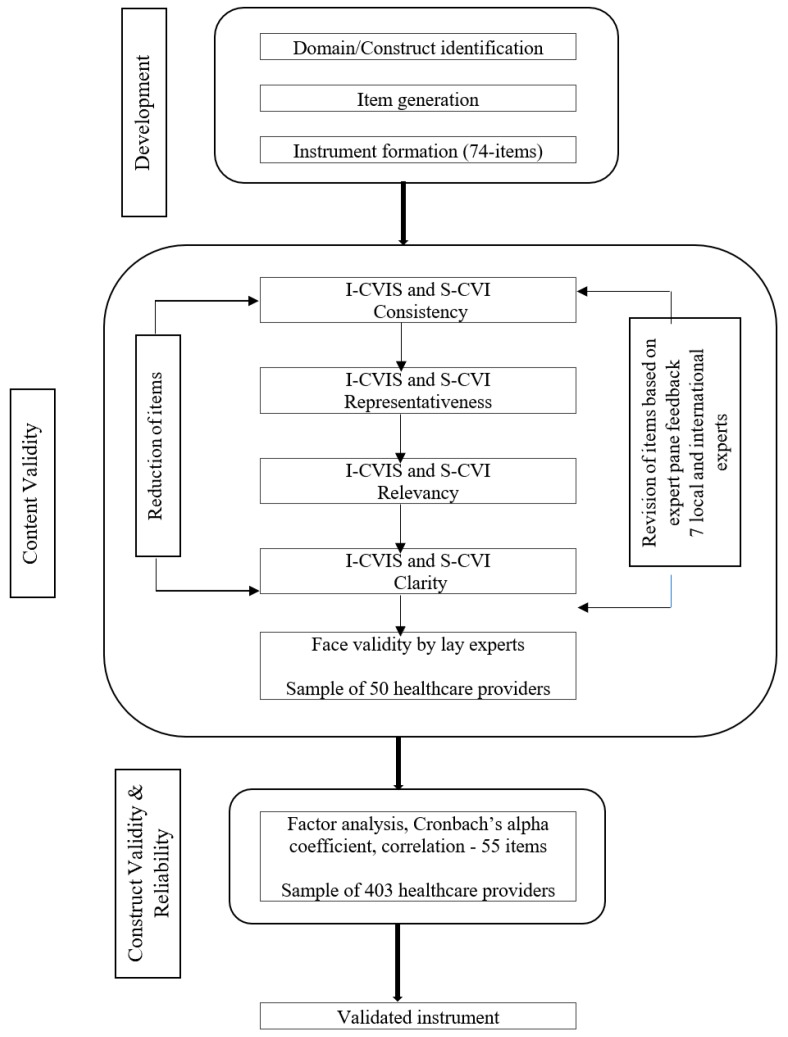
Steps of validity process.

The ProSCiTE development and validation involved three processes, including ProSCiTE development, content validity by expert panels, as well as construct validity and reliability through a pilot study among a sample of healthcare providers. A flowchart of the validation process is presented in Figure 1. 

### 2.1. ProSCiTE Development

The development of the “ProSCiTE” survey questionnaire was performed through three steps: determining the content domain/construct, sampling from the content (item generation), and instrument formation [57,58]. The questionnaire was adopted from previous studies [1,2,3,4,5,6,10,54,55,56,59] and was adapted to this study. 

An initial 74 items reflecting five constructs were developed using a conceptual model of how trainings might work, based on a combination of existing studies and the Integrated Change Model (I-Change Model) [60]. We studied healthcare providers’ behavioral changes at work with the 5 As method using the I-Change Model as a theoretical framework. Covertness and overtness are determined by a person’s motivation (attitudes, social influence, and self-efficacy) or intention to carry out behavior as a result of a person’s intentions and abilities. Intentions can range from not contemplating behavioral change to contemplating changes in behavior very rapidly, e.g., within a month. A person’s abilities, such as being able to plan specific actions to reach the behavior goal, and intentions to implement these actions (as well as actual skills) increase the chances of changing intentions into actions, while physical barriers can lower these chances [60].

Our conceptual framework assumes that healthcare providers’ sociodemographic characteristics, healthcare system-related factors, and patient-related factors might influence their level of knowledge, attitude, and self-efficacy. These factors further influence behavioral changes among healthcare providers toward smoking cessation interventions, which finally impacts the outcome of the smoking cessation provision among healthcare providers. Nevertheless, a total of 67 items that reflected the constructs were primarily approved by a local and international panel of experts on tobacco control. However, considering the potential response burden for participation, only constructs and items that had the greatest potential for predictive power and specificity for use in program planning and evaluation were included in this tool. This tool included a demographic background and five constructs: knowledge, attitude, self-efficacy, behavior, and barriers to providing smoking cessation interventions. Details of the initial development of constructs and items are illustrated in Table 1.

### 2.2. Content and Face Validity

#### 2.2.1. Participants and Setting

The content validation study was carried out over a three-month period from March to May 2016. International and local expert panels in the tobacco control field were invited to participate in the present study. Our interdisciplinary panel of experts was composed of two behavioral science specialists, one public health intervention specialist, two public health specialists, one dental public health specialist, and one consultant on pulmonary and critical care medicine. The experts were purposely selected to provide diverse ideas based on their specializations. They were selected according to the methodology described in Grant et al. considering their relevant training, experience, and qualifications as content experts. A history of publications in referred-to journals, local and national presentations, clinical expertise, and research on phenomena of interest were also regarded as criteria in selecting the content experts [61]. Zamzadeh (2015) has recommended that the number of experts be at least five to avoid chance agreement. A maximum of 10 experts was required in this study, as an increasing number of experts would decrease the probability of chance agreement [57]. A pilot test was conducted using 50 target populations to verify the tool’s feasibility. They were also requested to give their opinion and recommendation on the evaluation tool.

#### 2.2.2. Quantification of Content Validity

The expert panels were approached through email or self-distributed materials and were provided with an introductory cover letter, a set of evaluation tools, and an informed consent form. The expert panels were asked to evaluate based on four aspects (i.e., consistency, representativeness, relevancy, and clarity) for each item of the ProSCiTE. Each item of the questionnaire was ranked on a four-point scale (1 = not relevant, 2 = somewhat relevant, 3 = quite relevant, and 4 = highly relevant). The expert panels were also asked to provide recommendations to improve the sentence or structure based on difficulties they faced during the evaluation of the tool. The completed evaluation tool was returned to the researcher via the same medium.

### 2.3. Construct Validity and Reliability

#### 2.3.1. Participants and Setting

A cross-sectional study was carried out over a five-month period from July to December 2016. In this phase, only 55 out of 67 items from the content validity results were used in the exploratory factor analysis (EFA). These items concerned four constructs: attitudes, self-efficacy, behavior, and barriers to smoking cessation interventions. The knowledge domain could not be factor-analyzed due to different formats of response. Here, 403 healthcare providers (e.g., doctors, nurses, pharmacists, medical assistants, and health education officers) completed the paper-and-pencil questionnaire during the face-to-face study. The selected healthcare providers attended a smoking cessation training program called “Smoking Cessation Training Organizing, Planning, and Execution (SCOPE)”, as described in our published paper [62]. The data were collected before the start of the training program. Participation was voluntary. The sample size was determined based on the recommended minimum of five participants, as written by Tabachnick and Fidell (2013), for each questionnaire item for factor analysis [63]. The calculated sample size of 55 items representing 7 respondents for each item was 385. However, there were 403 actual respondents. Therefore, the total sample exceeded the minimum required sample size to analyze the ProSCiTE factor structure. The following were the inclusion criteria: (1) being a healthcare provider; (2) providing informed consent; (3) being able to comprehend the Malay/English language questionnaire; and (4) never having attended a SCOPE training. The exclusion criteria were foreign healthcare providers.

After explaining the study objectives and handling over the explanatory statements, the participants signed the informed consent forms. The researchers distributed the ProSCiTE tool among the healthcare providers and collected the tool upon completion. On average, approximately 15 to 30 min were needed to complete the ProSCiTE tool.

#### 2.3.2. Reliability

A determination of reliability was evaluated based on interitem correlation, Cronbach’s α, the internal consistency of the responses for each scale, and the entire instrument. Internal consistency reflects the extent to which questionnaire items are intercorrelated or whether they are consistent in the measurement of the same domain [64]. Charter (1999) has stated that a minimum sample size of 400 is a sufficiently precise estimate of the population coefficient alpha (α) [65].

#### 2.3.3. Determination of Correlation

Correlation was evaluated based on a Pearson correlation to determine the relationship between each domain. 

### 2.4. Ethical Approval

This study was approved by the Ministry of Health of Malaysia (Reference Number: NMRR-16-2144-32353 (IIR)) and the University of Malaya Medical Ethics Committee (Reference Number: UM. TNC2/RC/H&E/UMREC-118).

### 2.5. Statistical Methods

Statistical analyses were performed using IBM SPSS software (version 23.0 for Windows, SPSS, Inc., Chicago, IL, USA). All significant results were based on *p*-value < 0.05. 

#### 2.5.1. Descriptive Analysis

Descriptive analysis was used to measure the demographic characteristics of the healthcare providers. 

#### 2.5.2. Content Validity Analysis

The Content Validity Index (CVI) is extensively used in reporting the content validity of newly developed instruments [58,66,67,68,69,70,71,72]. In this study, the content validity of the developed instrument was statistically analyzed by an Item-Content Validity Index (I-CVI), a Scale-Content Validity Index (S-CVI), and kappa (*k*). The I-CVI for each item was calculated by dividing the number of experts who provided a rank of 3 or 4 by the total number of experts, and the S-CVI or average for each scale was calculated by summing the I-CVI divided by the number of items. According to Polit et al. (2006) [71], items that present an I-CVI score of >0.7 and an S-CVI minimum of 0.8 are considered to be acceptable for a new instrument. The kappa modified coefficient was used to determine the degree of relevance agreement of the CVIs and was calculated according to Polit et al. The evaluation of kappa values was done based on guidelines described in Cicchetti’s work: Fair = *k* of 0.4 to 0.59; Good = *k* of 0.6–0.74, and Excellent = *k* > 0.74 [73].

#### 2.5.3. Construct Validity Analysis

Exploratory factor analysis (EFA) using principal factor analysis with varimax rotation was employed to extract the underlying factor structure of the items. The Kaiser–Meyer–Olkin (KMO) test for sampling adequacy and Bartlett’s test of sphericity were examined before proceeding with further analyses. The KMO test reports the amount of variance in the data that can be explained by the factors. Values of 0.5 or lower are unacceptable and values of 0.6 or above are acceptable. Bartlett’s test of sphericity also indicates the suitability of the data for factor analysis [74]. A significant Bartlett’s test of *p* < 0.05 is considered acceptable. The factor loadings values were evaluated according to Comrey and Lee (1992): >0.32 = Poor, >0.45 = Fair, >0.55 = Good >0.63 = Very Good, and >0.7 = Excellent [75]. However, given the large sample size, a factor loading ≥0.38 was considered an appropriate cut-off point, and the Kaizer criterion was used to retain components with eigenvalues >1 [76].

#### 2.5.4. Reliability Analysis

Internal consistency was indexed by Cronbach’s alpha coefficient with 95% confidence intervals using Kistner and Muller’s *F* approximation [77]. The alpha that was the result of dropping each item from the domain was examined to see if the loss of any item increases the domain’s internal consistency, suggesting that the item did not contribute to the domain. The alpha values were evaluated based on Sekaran (2000): <0.6 = Poor, >0.7 = Acceptable, and >0.8 = Good [78]. 

#### 2.5.5. Correlation Analysis

A Pearson correlation of each domain with total scores was conducted to examine the relationship between each domain. Low correlation ranges were from 0.1 to 0.3, moderate ranges were from 0.3 to 0.5, and high ranges were >0.5 [79,80].

## 3. Results

### 3.1. Content and Face Validity

#### 3.1.1. Demographics of Expert Reviewers

Seven expert panels in the tobacco control field participated in this content validation. Almost equal numbers of them were male/female and local/international. All of them had more than 10 years of experience in the tobacco control field (Table 2).

#### 3.1.2. Content Validity

The original version of the ProSCiTE contained 74 items. However, after going through the content validation process with the expert reviewers, we decided to remove seven items from the behavior domain. Thus, the final version of the ProSCiTE contained 67 items. Table 3 shows the content validity for each domain. All items in the “Knowledge” domain demonstrated excellent content validity for the characteristics of consistency, representativeness, relevance, and clarity (CVI = 0.86–1.00 and kappa = 0.85–1.00). Thus, all 12 items in this domain were maintained. All items in the “Attitude” domain demonstrated excellent content validity for the characteristics of consistency, representativeness, relevance, and clarity (CVI = 0.85–1.00 and kappa = 0.86–1.00). Thus, all eight items in this domain were maintained. Most items in the “Behavior” domain obtained satisfactory scores. Item number 6a had its wording changed to better adapt it to the evaluation of behavior because it had low CVI and kappa values for all characteristics. Items 6b–6e were excluded from the tool due to low CVI and kappa values for most of the characteristics analyzed. Regarding item numbers 18, 19, and 20, even though all items had satisfactory scores, they were excluded because the questions were only suitable for psychiatrists. Apart from that, all items in the “Barriers” domain obtained satisfactory CVI and kappa values. Thus, all 15 items in this domain were maintained.

### 3.2. Construct Validity and Reliability

#### 3.2.1. Healthcare Providers’ Characteristics

Out of all the healthcare providers who participated in this study, 403 of them completed the survey, yielding a 90.0% response rate. The majority of the healthcare providers were female, Malay, and Muslim. Slightly above one third (35.2%) of the healthcare providers were pharmacists. There was an approximately equal proportion of healthcare providers aged 33 years old and below and above 33 years old. Almost half of them obtained a bachelor’s degree qualification. More than half (61.3%) of the healthcare providers had more than nine years of working experience. The majority (85.1%) of the healthcare providers reported that they were nonsmokers. More than half of the healthcare providers reported having experience in smoking cessation training, and slightly above half were interested in increasing their smoking cessation skills (see Table 4).

#### 3.2.2. Exploratory Factor Analysis

The ProSCiTE tool contained 55 items concerning attitudes, self-efficacy, behavior, and barriers to smoking cessation interventions for the factor analysis. In our study, one factor was clearly distinguished from another after the items underwent the extraction sum of squared loadings and then were rotated through varimax rotation with the Kaiser normalization method using SPSS (Table 5). The percentage of explained variance of the four factors was 51.68% of the total variance, which is a good structure. The sample adequacy of the ProSCiTE data for the EFA was confirmed based on its very good value (0.94) in the Kaiser–Meyer–Olkin test and its significant value in Bartlett’s test (*p* < 0.001). Hence, these values determined the appropriateness and sampling adequacy of the data for factor analysis [81].

#### 3.2.3. Item Reliability

Cronbach’s alpha was calculated to examine the internal consistency reliabilities of all the variables. The selections of items for each factor were scrutinized, and the combination of items with the largest α was retained (α should be at least 0.5) [64]. The alpha coefficient for the four constructs ranged from 0.89 to 0.96, which is considered to be “good to excellent” (Table 6). 

#### 3.2.4. Correlation Analysis

Table 7 shows the results of the correlation analysis using the Pearson coefficient (*r*) between all the construct scores in the total sample of participants. The results indicated that all constructs were positive and significantly correlated with each other, except for the association between barriers. The results demonstrated high item domain scores, particularly between self-efficacy and behavior (0.61), which may suggest that higher self-efficacy contributes to positive behavior in smoking cessation interventions. Moderate correlations were identified between attitudes and behavior (0.34), as well as between attitudes and self-efficacy (0.27).

## 4. Discussion

In any research or program evaluation effort, it is important to ensure that the outcomes of interest are clearly defined and that the outcomes are evaluated using valid and reliable measures. Thus, we developed and validated this study using a factor analysis technique for ProSCiTE, which is an evaluation tool for smoking cessation interventions among healthcare providers in Malaysia. The ProSCiTE is also salient in assessing and monitoring if staff training regarding smoking cessation leads to increased smoking cessation-related knowledge, more positive attitudes toward treating smokers, and increased self-efficacy in smoking cessation interventions. Thus, this tool is promising for evaluating the effectiveness of training in future studies that could also assess whether or not healthcare providers deliver evidence-based smoking cessation interventions over time. 

In this study, the development of the ProSCiTE was based on a broad literature review and existing scales. Validity and reliability were used to validate the ProSCiTE. The validity of a questionnaire is determined by analyzing if the questionnaire measures what it is intended to measure. In testing the validity, two types of validities were examined: content validity and construct validity. Content validity refers to the extent to which the items in a questionnaire are representative of the entire theoretical domain the questionnaire is designed to assess [82]. The ProSCiTE underwent face and content validity through seven local and international experts in the tobacco control field, who were selected based on their experience, knowledge, time, and willingness to participate in the process, apart from their effective communication skills as well as their publications and presentations. The I-CVI, modified kappa results, and comments from experts added rigor to the validation process of the ProSCiTE’s content. During the content validation phase, 67 items were successfully validated by the expert panels. Four items on assessing patients’ readiness to quit and three items on bupropion treatment were excluded from further analysis. These items may be relevant to psychiatrists but are not to general healthcare providers. In Malaysia, our standard practice is that only psychiatrists can prescribe bupropion to their patients, including for smoking cessation treatment.

Construct validity is important to measure a construct that is not directly observable. Therefore, in this study, attitude, self-efficacy, behavior, and barriers were measured by construct validity. The interpretation of results and inferences could not be drawn if the questionnaire lacked construct validity. In testing construct validity, a pilot study among healthcare providers was conducted to derive the main factors for smoking cessation training outcomes. Thus, an EFA with varimax rotation was performed to reduce the factors. An EFA was chosen due to the fact that this was the first validity test for a newly developed tool: we needed to measure if relationships existed between the variables, and what those were was not known earlier. A reliability test refers to the consistency of the results drawn from the questionnaire, which can be evaluated using its internal consistency, test–retest reliability, and inter-rater reliability. For the reliability test, an internal consistency was performed, which reflects the extent to which questionnaire items are intercorrelated or whether or not they are consistent in the measurement of the same domain using Cronbach’s alpha [64].

The construct validity and reliability tests (based on a survey of 403 healthcare providers) indicated that the ProSCiTE has good psychometric properties. The results of the EFA extracted four factors accordingly. All subscales demonstrated excellent internal consistency. This is the first study to develop and validate an evaluation tool for smoking cessation training in order to measure knowledge, attitude, self-efficacy, behavior, and barriers in Malaysia.

The main highlight of this research is that the reliability analysis of the four constructs showed excellent internal consistency, as indicated by Cronbach’s alpha. This study reported a Cronbach’s alpha minimum of 0.89 and a maximum of 0.95 for four of the domains. The value of alpha > 0.7 verified that all items in the factor were highly correlated with each other, providing assurance that random errors were minimized in the factor [72]. One similar study that included other additional domains found an α-value ranging from 0.82 to 0.93 [1]. The EFA result in the four domains from this study showed some similarity with previous studies [1,2,5]. Healthcare providers understood their role of advising and assisting patients to stop using any tobacco products, but they failed to understand their patients’ needs in terms of helping them to quit. Our finding for the barrier item “patients not interested” was slightly higher compared to a previous study [2].

In addition, our results for the correlation for each construct were comparable to previous studies [1,2]), except for the barriers construct. Though in the present study we found that barriers were positively correlated with knowledge, attitude, and behavior (compared to previous studies), the correlation was very weak. However, our finding on the significant negative correlation between barriers and self-efficacy was parallel to the finding by Zapka et al. [1], indicating that healthcare providers with high barriers may have lower self-efficacy in providing smoking cessation interventions. Zapka et al. [1] also found that barriers were negatively significantly correlated with the performance of smoking cessation interventions. The differences warrant further investigations, especially using qualitative approaches. They could be explained by the perceptions of healthcare providers toward barriers, as the barriers in the tool cover all aspects of patients, healthcare providers, and the system.

To the best of our knowledge, the ProSCiTE is the first published study that has been developed and that has undergone a detailed validation tailored for smoking cessation training. The involvement of selected expert panels based on their expertise in training and tobacco control could strengthen the face and content validity of the ProSCiTE. Although the 67 questions included in the ProSCiTE seem like a large number and might result in a long completion time, the ProSCiTE is able to detect various impacts of a training from multiple aspects. Since most of the currently available measures emphasize particular aspects such as knowledge, attitudes, and practices, the ProSCiTE can assess the impacts that are not detected by existing measures, including the withdrawal symptoms of smokers. Healthcare providers need to understand the withdrawal symptoms of their patients in order for them to prescribe their patients either pharmacological or behavioral treatments, dealing with withdrawal symptoms as well as other issues related to quitting. This would help healthcare providers understand their patients’ level of addiction and detect changes caused by treatment, as well as allow them to choose the best nicotine replacement therapy products.

Furthermore, the criteria validity of the ProSCiTE has not been validated due to the fact that there is no published gold standard for the evaluation of smoking cessation trainings for a comparison to ProSCiTE. While the ProSCiTE was under development, several research works were published on the measurement of smoking cessation that focused on knowledge of health consequences, attitudes, self-efficacy, practices, and barriers [1,2,3,5,6,7,8]. However, the number of items used in each construct, the rating scales, and the scoring were different, and thus they cannot be directly compared to the ProSCiTE.

This study successfully validated an evaluation tool, ProSCiTE for Malaysian healthcare providers, based on the 5 As of smoking cessation interventions. However, since the ProSCiTE does not contain items that are specifically related to Malaysian culture, it could be used internationally to evaluate the effectiveness of any smoking cessation training program based on the 5 As. The ProSCiTE was developed through adaption from and the adoption of previous literature on cognitive, behavioral, individual, clinical, and organizational factors [6,51,52,83,84]. We found that all of the factors seemed to be important for behavior regarding smoking cessation interventions, particularly attitude and self-efficacy. Thus, we can use the ProSCiTE to measure the magnitude of construct changes following smoking cessation training. We postulate that the changes may affect clinical practice for smoking cessation interventions in terms of the number of patients counseled using evidence-based smoking cessation treatments. This treatment could increase the effectiveness of the intervention, which leads to increasing numbers of patients who quit smoking. 

Nevertheless, our study had some limitations that should be addressed. First, the major limitations that this tool has are that this tool is mainly directed toward evaluating training perceptions among healthcare providers (doctors, pharmacists, medical assistants, health education officers, nurses, and others) receiving basic or level I SCOPE training [62]. Second, it relies on self-reported responses from healthcare providers; thus, recall bias, social desirability bias, and information bias should be addressed. There should be careful consideration, as healthcare providers tend to over-report the frequency and actual performance of the 5 As smoking cessation intervention [42,85]. However, the anonymous nature of responses and the fact that respondents knew that their data were confidential reduced this bias. Furthermore, the questionnaire developed in this study was asked in both a positive and negative manner, was adopted and adapted from previous literature, and was validated by expert panels [86]. Our study consisted of heterogenized samples of healthcare providers with different roles and responsibilities in the healthcare setting, which is in line with the World Health Organization (WHO)’s Free Initiative. The initiative recommends that smoking cessation practices not be exclusive to one professional group and should be provided by all healthcare providers [87]. More objective monitoring based on patients’ medical charts and direct observation of the patients’ outcomes might improve and strengthen this finding. Third, our evaluation of knowledge was specific to withdrawal symptoms for smoking cessation, compared to other instruments that focus on health consequences. The assessment of knowledge may vary based on the content of the training program and guideline use in each country. Therefore, a standardized tool is difficult to use for any other trainings. Lastly, long-term effectiveness with controlled pre- and post-training to measure changes over time should be continuously evaluated in order to improve and validate the tool in accordance with current practices. Future research should investigate additional psychometric testing such as confirmatory factor analysis. Criterion validity should also be measured.

## 5. Conclusions

In conclusion, assessments of the content, construct, and internal consistency reliability of an evaluation tool are critical to obtain valid and reliable research findings. Our ProSCiTE tool exhibited good content validity, construct validity, and internal consistency reliability. It also offers a starting point for further tool development and a measure for evaluating tobacco-related policy and training programs. The ProSCiTE also measures the delivery of the 5 As intervention as an innovative evaluation tool for researchers, managers, and practitioners. They can utilize this promising newly validated tool to evaluate their smoking cessation-related programs.

## Figures and Tables

**Table 1:** Initial development of constructs and items in the Providers’ Smoking Cessation Training Evaluation (ProSCiTE).

Construct	Operational Definition	Items	Responses
Knowledge	Knowledge is information and understanding that you get from experience or education.	1. Irritability 2. Depression 3. Restlessness 4. Poor concentration 5. Increased appetite 6. Weight gain 7. Lightheadedness 8. Nighttime awakening 9. Constipation 10. Diarrhea 11. Mouth ulcers 12. Urge to smoke	0 = No 1 = Yes
Attitude	Attitude is the tendency, based on trust and experience, to respond to a smoking cessation intervention with specific methods and approaches.	1. A patient’s chance of quitting smoking increases if the healthcare provider advises him/her to quit. 2. Patients want you to advise them to stop using any tobacco products. Healthcare providers like you should… 3. get specific training on smoking cessation counseling techniques. 4. set a good example for their patients and public by not using any tobacco products. 5. routinely ask patients about tobacco use. 6. routinely ask parents/guardians about tobacco use during pediatric visits. 7. routinely advise patients who use any tobacco products to quit. 8. routinely assist patients using any tobacco products to quit.	1 = Strongly disagree 2 = Disagree 3 = Neither disagree/agree 4 = Agree 5 = Strongly agree
Self-efficacy	Self-efficacy is one’s belief in one’s ability to succeed in specific situations or accomplish a task in a smoking cessation intervention. Self-efficacy in this study refers to the confidence to provide a smoking cessation intervention using the 5 As brief intervention for smoking cessation.	1. I know appropriate questions to ask my patients. 2. I am able to motivate my patients who are interested in quitting smoking. 3. I am able to assist patients to quit even if the patient thinks that it is difficult to give up. 4. I have the pharmacological therapy skills to assist patients to quit smoking. 5. I have the behavioral therapy skills to assist patients to quit smoking. 6. I can advise patients to consider smoking cessation. 7. I can provide counseling when time is limited. 8. I can counsel patients who are not interested in quitting. 9. I know how to prescribe medication (nicotine replacement therapy/bupropion) to treat tobacco dependency. 10. I can assess a patient’s different stages of readiness to quit smoking. 11. I can assess a patient’s level of nicotine dependency using the Fagerstrom test. 12. I can use a smokerlyzer to determine a patient’s carbon monoxide level. 13. I can assist recent quitters to learn how to cope with situations or triggers that might lead them to relapse to using tobacco.	1 = Certainly not 2 = Probably not 3 = Neutral 4 = Probably 5 = Certainly
Behavior	Behavior is the way in which healthcare providers act in response to any particular situation or stimulus regarding smoking cessation interventions. Behavior in this study refers to the delivery of the 5 As brief intervention for smoking cessation.	In your current practice, how often do you… 1. ask patients whether they smoke? 2. ask patients the number of cigarettes smoked per day? 3. advise patients who smoke to quit smoking? 4. advise female patients to quit smoking if they are pregnant or planning to become pregnant? 5. advise patients to quit smoking if you think their illness is related to smoking? 6. assess patients’ readiness to quit smoking according to the stages of change below? a. precontemplation b. contemplation c. preparation d. action e. maintenance 7. assess reasons for quitting/continuing to quit smoking? 8. assist those who are not interested in quitting smoking to think about quitting? 9. assist those who are interested in quitting smoking to develop a plan to quit? 10. assist in setting quitting dates? 11. arrange referrals for appropriate smoking cessation services? 12. provide counseling for patients who want to quit smoking? 13. provide educational materials related to smoking cessation? 14. document tobacco-relevant discussions and plans in medical records? 15. use the Fagerstrom test to assess a patient’s level of addiction? 16. use a smokerlyzer to determine a patient’s carbon monoxide level? 17. prescribe or recommend the purchase of nicotine replacement therapy products for patients attempting to quit? 18. prescribe bupropion to those ready to quit smoking? (if applicable) 19. prescribe a combination of bupropion and nicotine replacement products to those ready to quit smoking? (if applicable) 20. prescribe second-line medications (e.g., clonidine, nortriptyline, bupropion) to patients ready to quit smoking? (if applicable) 21. provide treatment maintenance and follow-up services to those who have quit smoking? 22. arrange a follow-up visit or phone call to discuss quitting smoking?	1 = Never 2 = Rarely 3 = Sometimes 4 = Often 5 = Always
Barrier	Barrier is a law, rule, or problem that makes something difficult or impossible or that might limit or prevent the capacity to offer a smoking cessation intervention for patients using the 5 As brief intervention for smoking cessation.	1. Patients not interested in quitting smoking. 2. Patients not ready to change. 3. Patients do not comply with the given pharmacological therapy. 4. Patients do not comply with the given behavioral therapy. 5. Lack of impact of pharmacological therapy on patients. 6. Lack of impact of behavioral therapy on patients. 7. Lack of knowledge on smoking cessation. 8. Lack of time. 9. Other health problems require priority treatment. 10. Lack of reimbursement to healthcare providers. 11. Lack of community resources to which to refer patients. 12. Inadequate smoking cessation pharmaceutical drugs. 13. Lack of patient education materials. 14. Lack of smoking cessation training. 15. Complexity of smoking cessation guidelines.	1 = Not a barrier 2 = Somewhat a barrier 3 = Moderate barrier 4 = Extreme barrier

**Table 2:** Demographics of expert reviewers.

Variable	Category	Frequency (*n* = 7)	Percentage (%)
**Gender**	Male	4	57.14
	Female	3	42.86
**Region**	Local	3	42.86
	International	4	57.14
**Experience in tobacco control field**	>10 years	7	100.00

**Table 3:** Content validity of the ProSCiTE constructs.

Constructs	Items	Consistency		Representativeness		Relevancy		Clarity		Results
		I-CVI *	*k*	I-CVI *	*k*	I-CVI *	*k*	I-CVI *	*k*	
**Knowledge**	1. Irritability	1	1	1	1	1	1	1	1	VALIDATED
	2. Depression	1	1	1	1	1	1	1	1	VALIDATED
	3. Restlessness	1	1	1	1	1	1	1	1	VALIDATED
	4. Poor concentration	1	1	1	1	1	1	1	1	VALIDATED
	5. Increased appetite	1	1	1	1	1	1	1	1	VALIDATED
	6. Weight gain	1	1	1	1	1	1	1	1	VALIDATED
	7. Lightheadedness	0.86	0.85	0.86	0.85	0.86	0.85	0.86	0.85	VALIDATED
	8. Nighttime awakening	1	1	1	1	1	1	1	1	VALIDATED
	9. Constipation	1	1	1	1	1	1	1	1	VALIDATED
	10. Diarrhea	1	1	1	t	1	1	1	1	VALIDATED
	11. Mouth ulcers	1	1	1	1	1	1	0.86	0.85	VALIDATED
	Item 12	0.86	0.85	0.86	0.85	0.86	0.85	0.86	0.85	VALIDATED
	S-CVI	0.98		0.98		0.98		0.97		
**Attitude**	1. A patient’s chance of quitting smoking increases if the healthcare provider advises him/her to quit.	1	1	1	1	1	1	1	1	VALIDATED
	2. Patients want you to advise them to stop using any tobacco products.	0.86	0.85	0.86	0.85	0.86	0.85	0.86	0.85	VALIDATED
	Healthcare providers like you should…									
	3. get specific training on smoking cessation counseling techniques.	1	1	1	1	1	1	1	1	VALIDATED
	4. set a good example for their patients and public by not using any tobacco products.	1	1	1	1	1	1	1	1	VALIDATED
	5. routinely ask patients about tobacco use.	1	1	1	1	1	1	1	1	VALIDATED
	6. routinely ask parents/guardians about tobacco use during pediatric visits.	1	1	1	1	1	1	1	1	VALIDATED
	7. routinely advise patients who use any tobacco products to quit.	1	1	1	1	1	1	0.86	0.85	VALIDATED
	8. routinely assist patients using any tobacco products to quit.	1	1	1	1	1	1	0.86	0.85	VALIDATED
	S-CVI	0.98		0.98		0.98		0.95		
**Self-efficacy**	1. I know appropriate questions to ask my patients.	1	1	0.57	0.41	1	1	1	1	VALIDATED
	2. I am able to motivate my patients who are interested in quitting smoking.	1	1	0.71	0.66	1	1	1	1	VALIDATED
	3. I am able to assist patients to quit even if the patient thinks that it is difficult to give up.	1	1	0.71	0.66	1	1	0.86	0.85	VALIDATED
	4. I have the pharmacological therapy skills to assist patients to quit smoking.	1	1	0.57	0.41	1	1	1	1	VALIDATED
	5. I have the behavioral therapy skills to assist patients to quit smoking.	1	1	0.71	0.66	1	1	1	1	VALIDATED
	6. I can advise patients to consider smoking cessation.	0.86	0.85	0.57	0.41	0.71	0.66	1	1	VALIDATED
	7. I can provide counseling when time is limited.	1	1	1	1	1	1	0.86	0.85	VALIDATED
	8. I can counsel patients who are not interested in quitting.	1	1	1	1	0.86	0.85	1	1	VALIDATED
	9. I know how to prescribe medication (nicotine replacement therapy/bupropion) to treat tobacco dependency.	0.86	0.85	1	1	1	1	1	1	VALIDATED
	10. I can assess a patient’s different stages of readiness to quit smoking.	1	1	1	1	0.86	0.85	1	1	VALIDATED
	11. I can assess a patient’s level of nicotine dependency using the Fagerstrom test.	1	1	1	1	1	1	1	1	VALIDATED
	12. I can use a smokerlyzer to determine a patient’s carbon monoxide level.	1	1	0.86	0.85	1	1	1	1	VALIDATED
	13. I can assist recent quitters to learn how to cope with situations or triggers that might lead them to relapse in using tobacco.	1	1	1	1	1	1	1	1	VALIDATED
	S-CVI	0.98		0.82		0.86		0.98		
**Behavior**	In your current practice, how often do you….									
	1. ask patients whether they smoke?	1	1	1	1	1	1	1	1	VALIDATED
	2. ask patients the number of cigarettes smoked per day?	1	1	1	1	1	1	1	1	VALIDATED
	3. advise patients who smoke to quit smoking?	1	1	1	1	1	1	1	1	VALIDATED
	4. advise female patients to quit smoking if they are pregnant or planning to become pregnant?	1	1	1	1	1	1	1	1	VALIDATED
	5. advise patients to quit smoking if you think their illness is related to smoking?	1	1	1	1	1	1	1	1	VALIDATED
	6. assess a patient’s readiness to quit smoking according to the stages of change below?									CORRECTED
	a. precontemplation	0.71	0.66	0.71	0.66	0.57	0.41	0.71	0.66	EXCLUDED
	b. contemplation	0.71	0.66	0.71	0.66	0.57	0.41	0.71	0.66	EXCLUDED
	c. preparation	0.57	0.41	0.57	0.41	0.43	0.21	0.57	0.41	EXCLUDED
	d. action	0.57	0.41	0.57	0.41	0.43	0.21	0.57	0.41	EXCLUDED
	e. maintenance	0.57	0.41	0.57	0.41	0.43	0.21	0.57	0.41	EXCLUDED
	7. assess reasons for quitting/continuing to quit smoking?	0.71	0.66	0.86	0.85	0.86	0.85	0.86	0.85	VALIDATED
	8. assist those who are not interested in quitting smoking to think about quitting?	1	1	1	1	1	1	0.86	0.85	VALIDATED
	9. assist those who are interested in quitting smoking to develop a plan to quit?	1	1	1	1	1	1	0.86	0.85	VALIDATED
	10. assist in setting quitting dates?	1	1	1	1	1	1	1	1	VALIDATED
	11. arrange referrals for appropriate smoking cessation services?	1	1	0.86	0.85	1	1	1	1	VALIDATED
	12. provide counseling for patients who want to quit smoking?	0.86	0.85	0.86	0.85	1	1	0.86	0.85	VALIDATED
	13. provide educational materials related to smoking cessation?	1	1	1	1	1	1	1	1	VALIDATED
	14. document tobacco-relevant discussions and plans in medical records?	1	1	1	1	1	1	1	1	VALIDATED
	15. use the Fagerstrom test to assess a patient’s level of addiction?	1	1	1	1	1	1	1	1	VALIDATED
	16. use a smokerlyzer to determine a patient’s carbon monoxide level?	0.86	0.85	1	1	1	1	1	1	VALIDATED
	17. prescribe or recommend the purchase of nicotine replacement therapy products for patients attempting to quit?	1	1	1	1	1	1	1	1	VALIDATED
	18. prescribe bupropion to those ready to quit smoking? (if applicable)	1	1	1	1	1	1	1	1	EXCLUDED
	19. prescribe a combination of bupropion and nicotine replacement products to those ready to quit smoking? (if applicable)	0.86	0.85	0.86	0.85	0.86	0.85	0.86	0.85	EXCLUDED
	20. prescribe second-line medications (e.g., clonidine, nortriptyline, bupropion) to patients ready to quit smoking? (if applicable)	0.86	0.85	0.86	0.85	0.86	0.85	0.86	0.85	EXCLUDED
	21. provide treatment maintenance and follow-up services to those who have quit smoking?	1	1	1	1	1	1	1	1	VALIDATED
	22. arrange a follow up visit or phone call to discuss quitting smoking?	1	1	1	1	1	1	1	1	VALIDATED
	S-CVI	0.90		0.90		0.89		0.90		
**Barriers**	1. Patients not interested in quitting smoking.	1	1	1	1	1	1	1	1	VALIDATED
	2. Patients not ready to change.	1	1	1	1	0.86	0.85	1	1	VALIDATED
	3. Patients do not comply with the given pharmacological therapy.	1	1	1	1	0.86	0.85	1	1	VALIDATED
	4. Patients do not comply with the given behavioral therapy.	1	1	1	1	0.86	0.85	1	1	VALIDATED
	5. Lack of impact of pharmacological therapy on patients.	1	1	1	1	1	1	1	1	VALIDATED
	6. Lack of impact of behavioral therapy on patients.	1	1	1	1	1	1	1	1	VALIDATED
	7. Lack of knowledge on smoking cessation.	1	1	1	1	1	1	1	1	VALIDATED
	8. Lack of time.	1	1	1	1	1	1	1	1	VALIDATED
	9. Other health problems require priority treatment.	1	1	1	1	1	1	1	1	VALIDATED
	10. Lack of reimbursement to healthcare providers.	0.86	0.85	0.86	0.85	0.86	0.85	1	1	VALIDATED
	11. Lack of community resources to which to refer patients.	1	1	0.86	0.85	0.86	0.85	1	1	VALIDATED
	12. Inadequate smoking cessation pharmaceutical drugs.	1	1	0.86	0.85	0.86	0.85	1	1	VALIDATED
	13. Lack of patient education materials.	1	1	1	1	1	1	1	1	VALIDATED
	14. Lack of smoking cessation training.	1	1	1	1	1	1	1	1	VALIDATED
	15. Complexity of smoking cessation guidelines.	1	1	1	1	1	1	1	1	VALIDATED
	S-CVI	0.99		0.97		0.94		1.0		

Item-Content Validity Index (I-CVI) = number of experts providing a rating of 3 or 4/the number of experts. Probability of chance agreement (Pc) = number of panelists who agreed that the item was relevant. Kappa (*k*) = kappa designating agreement on relevance. * Evaluation criteria for the level of content validity (relationship between I-CVI and *k*): excellent validity = I-CVI ≥ 0.78 and *k* > 0.74, good validity I-CVI < 0.78 and ≥0.60 and *k* ≤ 0.74, fair validity I-CVI < 0.6 and ≥0.40 and *k* ≤ 0.59, and poor validity I-CVI < 0.4 and *k* < 0.40. Scale-Content Validity Index (S-CVI) = sum of I-CVI/total items.

**Table 4:** Healthcare providers’ characteristics (*n* = 403).

Variables	Category	Frequency (*n*)	Percentage (%)
**Age**	<33 years old	238	59.1
	≥33 years old	165	40.9
**Gender**	Male	151	37.5
	Female	252	62.5
**Ethnicity**	Malay	270	67.0
	Chinese	93	23.1
	Indian	30	7.4
	Others	10	2.5
**Religion**	Islam	278	69.2
	Buddhist	66	16.4
	Christian	30	7.5
	Hindu	19	4.7
	Others	9	2.2
**Education level**	Diploma	124	30.8
	Bachelor	194	48.1
	Master	73	18.1
	PhD	2	0.5
	Others	10	2.5
**Profession**	Nurse	72	17.9
	Medical assistant	62	15.4
	Doctor	112	27.8
	Dentist	6	1.5
	Pharmacist	142	35.2
	HEO	4	1.0
	Others	5	1.2
**Smoking status ^a^**	Current smoker	14	3.5
	Former smoker	28	7.0
	Nonsmoker	358	89.5
**Working experience**	<9 years	247	61.3
	≥9 years	156	38.7
**Smoking cessation training**	Yes	175	43.4
	No	228	56.6
**Interest in upgrading smoking cessation skills ^b^**	Not very interested	17	4.30
	Not interested	15	3.80
	Somewhat interested	161	40.6
	Extremely interested	204	51.4

^a^*n* = 400; ^b^ *n* = 397; HEO: health education officer.

**Table 5:** Rotated factor pattern.

Construct	Items	Factor Loadings
**Attitude**	7. Healthcare providers like you should routinely advise patients who use any tobacco products to quit.	0.82
	8. Healthcare providers like you should routinely assist patients using any tobacco products to quit.	0.81
	3. Healthcare providers like you should get specific training on smoking cessation counseling techniques.	0.81
	4. Healthcare providers like you should set a good example for their patients and the public by not using any tobacco products.	0.80
	5. Healthcare providers like you should routinely ask patients about tobacco use.	0.77
	6. Healthcare providers like you should routinely ask parents/guardians about tobacco use during pediatric visits.	0.73
	1. A patient’s chance of quitting smoking increases if the healthcare provider advises him/her to quit.	0.48
	2. Patients want you to advise them to stop using any tobacco products.	0.40
**Self-efficacy**	13. I can assist recent quitters to learn how to cope with situations or triggers that might lead them to relapse to using tobacco.	0.82
	10. I can assess a patient’s different stages of readiness to quit smoking.	0.77
	5. I have the behavioral therapy skills to assist patients in quitting smoking.	0.77
	4. I have the pharmacological therapy skills to assist patients in quitting smoking.	0.76
	3. I am able to assist patients to quit even if the patient thinks that it is difficult to give up.	0.75
	9. I know how to prescribe medication (nicotine replacement therapy/bupropion) to treat tobacco dependency.	0.74
	11. I can assess a patient’s level of nicotine dependency using the Fagerstrom test.	0.73
	8. I can counsel patients who are not interested in quitting.	0.73
	7. I can provide counseling when time is limited.	0.71
	1. I know appropriate questions to ask my patients.	0.68
	2. I am able to motivate my patients who are interested in quitting smoking.	0.67
	12. I can use a smokerlyzer to determine a patient’s carbon monoxide level.	0.65
	6. I can advise patients to consider smoking cessation.	0.57
**Behavior**	In your current practice, how often do you…	
	10. assist in setting quit dates?	0.77
	7. assess reasons for quitting/continuing to quit smoking?	0.76
	9. assist those who are interested in quitting smoking to develop a plan to quit?	0.74
	6. assess patients’ readiness to quit smoking?	0.74
	11. arrange referrals for appropriate smoking cessation services?	0.74
	2. ask patients the number of cigarettes smoked per day?	0.73
	14. document tobacco-relevant discussions and plans in medical records?	0.73
	3. advise patients who smoke to quit smoking?	0.72
	12. provide counseling for patients who want to quit smoking?	0.71
	15. use the Fagerstrom test to assess a patient’s level of addiction?	0.70
	22. arrange a follow up visit or phone call to discuss quitting smoking?	0.68
	13 provide educational materials related to smoking cessation?	0.67
	8. assist those who are not interested in quitting smoking to think about quitting?	0.66
	1. ask patients whether they smoke?	0.66
	21. provide treatment maintenance and follow-up services to those who have quit smoking?	0.65
	16. use smokerlyzer to determine patient’s Carbon Monoxide level?	0.65
	5. advise patients to quit smoking if you think their illness is related to smoking?	0.64
	4. advise female patients to quit smoking if they are pregnant or planning to become pregnant?	0.58
	17. prescribe or recommend the purchase of nicotine replacement therapy products for patients attempting to quit?	0.55
**Barrier**	6. Lack of impact of behavioral therapy on patients.	0.72
	5. Lack of impact of pharmacological therapy on patients.	0.72
	7. Lack of knowledge on smoking cessation.	0.71
	14. Lack of smoking cessation training.	0.70
	11. Lack of community resources to which to refer patients.	0.68
	4. Patients do not comply with the given behavioral therapy.	0.67
	13. Lack of patient/client education materials.	0.67
	15. Complexity of smoking cessation guidelines.	0.66
	3. Patients do not comply with the given pharmacological therapy.	0.65
	9. Other health problems require priority treatment.	0.65
	12. Inadequate smoking cessation pharmaceutical drugs.	0.62
	8. Lack of time.	0.58
	10. Lack of reimbursement to healthcare providers.	0.57
	2. Patients not ready to change.	0.55
	1. Patients not interested in quitting smoking.	0.50

**Table 6:** Constructs and item statistics.

Construct	Items	*N*	Min.	Max.	Mean	SD	α
**Attitude**	1.A patient’s chance of quitting smoking increases if the healthcare provider advises him/her to quit.	403	1	5	3.93	0.82	0.89
	2. Patients want you to advise them to stop using any tobacco products.	403	1	5	3.70	0.86	
	Healthcare providers like you should…	403	1	5	4.51	0.68	
	3. get specific training on smoking cessation counseling techniques.	403	1	5	4.54	0.66	
	4. set a good example for their patients and public by not using any tobacco products.	403	1	5	4.26	0.74	
	5. routinely ask patients about tobacco use.	403	1	5	4.20	0.76	
	6. routinely ask parents/guardians about tobacco use during pediatric visits.	403	1	5	4.32	0.70	
	7. routinely advise patients who use any tobacco products to quit.	403	1	5	4.33	0.71	
	**Total**	**403**	**8**	**40**	**33.79**	**4.43**	
**Self-efficacy**	1. I know appropriate questions to ask my patients.	403	1	5	3.72	0.90	0.94
	2. I am able to motivate my patients who are interested in quitting smoking.	403	1	5	3.75	0.86	
	3. I am able to assist patients to quit even if the patient thinks that it is difficult to give up.	403	1	5	3.58	0.85	
	4. I have the pharmacological therapy skills to assist patients in quitting smoking.	403	1	5	3.38	1.09	
	5. I have the behavioral therapy skills to assist patients in quitting smoking.	403	1	5	3.23	1.03	
	6. I can advise patients to consider smoking cessation.	403	1	5	3.98	0.75	
	7. I can provide counseling when time is limited.	403	1	5	3.32	1.00	
	8. I can counsel patients who are not interested in quitting.	403	1	5	3.21	0.98	
	9. I know how to prescribe medication (nicotine replacement therapy/bupropion) to treat tobacco dependency.	403	1	5	3.07	1.21	
	10. I can assess a patient’s different stages of readiness to quit smoking.	403	1	5	3.34	1.07	
	11. I can assess a patient’s level of nicotine dependency using the Fagerstrom test.	403	1	5	3.28	1.27	
	12. I can use a smokerlyzer to determine a patient’s carbon monoxide level.	403	1	5	2.96	1.37	
	13. I can assist recent quitters to learn how to cope with situations or triggers that might lead them to relapse to using tobacco.	403	1	5	3.28	1.13	
	**Total**	**403**	**13**	**65**	**44.09**	**10.41**	
**Behavior**	In your current practice, how often do you….						
	1. ask patients whether they smoke?	403	1	5	3.64	0.98	0.96
	2. ask patients the number of cigarettes smoked per day?	403	1	5	3.46	1.04	
	3. advise patients who smoke to quit smoking?	403	1	5	3.80	0.99	
	4. advise female patients to quit smoking if they are pregnant or planning to become pregnant?	403	1	5	3.75	1.35	
	5. advise patients to quit smoking if you think their illness is related to smoking?	403	1	5	4.17	0.92	
	6. assess a patient’s readiness to quit smoking?	403	1	5	3.55	1.11	
	7. assess reasons for quitting/continuing to quit smoking?	403	1	5	3.53	1.06	
	8. assist those who are not interested in quitting smoking to think about quitting?	403	1	5	3.55	1.04	
	9. assist those who are interested in quitting smoking to develop a plan to quit?	403	1	5	3.57	1.11	
	10. assist in setting quit dates?	403	1	5	3.16	1.29	
	11. arrange referrals for appropriate smoking cessation services?	403	1	5	3.16	1.25	
	12. provide counseling for patients who want to quit smoking?	403	1	5	3.43	1.25	
	13. provide educational materials related to smoking cessation?	403	1	5	3.20	1.24	
	14. document tobacco-relevant discussions and plans in medical records?	403	1	5	2.94	1.35	
	15. use the Fagerstrom test to assess a patient’s level of addiction?	403	1	5	2.86	1.52	
	16. use a smokerlyzer to determine a patient’s carbon monoxide level?	403	1	5	2.45	1.52	
	17. prescribe or recommend the purchase of nicotine replacement therapy products for patients attempting to quit?	403	1	5	2.90	1.42	
	21. provide treatment maintenance and follow-up services to those who have quit smoking?	403	1	5	2.76	1.47	
	22. arrange a follow-up visit or phone call to discuss quitting smoking?	403	1	5	2.67	1.44	
	**Total**	**403**	**19**	**95**	**62.55**	**17.37**	
**Barrier**	1. Patients not interested in quitting smoking.	403	1	4	3.30	0.78	0.90
	2. Patients not ready to change.	403	1	4	3.30	0.77	
	3. Patients do not comply with the given pharmacological therapy.	403	1	4	3.00	0.80	
	4. Patients do not comply with the given behavioral therapy.	403	1	4	2.99	0.76	
	5. Lack of impact of pharmacological therapy on patients.	403	1	4	2.84	0.78	
	6. Lack of impact of behavioral therapy on patients.	403	1	4	2.89	0.73	
	7. Lack of knowledge on smoking cessation.	403	1	4	2.76	0.87	
	8. Lack of time.	403	1	4	2.83	0.87	
	9. Other health problems require priority treatment.	403	1	4	2.63	0.85	
	10. Lack of reimbursement to healthcare providers.	403	1	4	2.44	0.90	
	11. Lack of community resources to which to refer patients.	403	1	4	2.66	0.88	
	12. Inadequate smoking cessation pharmaceutical drugs.	403	1	4	2.68	0.92	
	13. Lack of patient education materials.	403	1	4	2.59	0.85	
	14. Lack of smoking cessation training.	403	1	4	2.86	0.83	
	15. Complexity of smoking cessation guidelines.	403	1	4	2.56	0.82	
	**Total**	**403**	**15**	**60**	**42.33**	**8.00**	

SD = standard deviation; α = Cronbach’s alpha.

**Table 7:** Bivariate correlations.

Constructs	Attitude	Self-Efficacy	Behavior	Barriers
**Attitude**	1			
**Self-efficacy**	0.27**	1		
**Behavior**	0.34**	0.61**	1	
**Barriers**	0.09	−0.04	0.08	1

** *p*-Value is significant at the 0.001 level.
